# Sex differences in associations between melatonin secretion, core body temperature, and cognitive function in older adults

**DOI:** 10.1002/alz70857_107057

**Published:** 2025-12-25

**Authors:** H. Matthew Lehrer, Aryan Govil, Hau‐Tieng Wu, Robert T Krafty, Meryl A Butters, Esther M Blessing, Daniel J Buysse

**Affiliations:** ^1^ University of Pittsburgh School of Medicine, Pittsburgh, PA, USA; ^2^ New York University, New York City, NY, USA; ^3^ Emory University, Atlanta, GA, USA

## Abstract

**Background:**

Nocturnal melatonin secretion may contribute to the nocturnal core body temperature (Tc) drop (Tdrop) and benefit cognitive function, which itself is affected by Tc. Prior research has not examined sex differences in melatonin's associations with Tc or cognition, impeding understanding of melatonin's potential pro‐cognitive actions. We tested sex differences in associations of melatonin area under the curve (AUC) and dim light melatonin onset (DLMO) with Tdrop and cognitive function in older adults.

**Method:**

Participants—65 cognitively unimpaired retired adults (32 postmenopausal females, 33 males; mean age: 68.4 +/‐5.6 years) —completed a 60‐hour laboratory study, including one night of baseline sleep followed by a 24‐hour circadian unmasking protocol including one night of sleep deprivation (SD), followed by a recovery night sleep (Recovery). Tc was continuously assessed with ingestible telemetry. Salivary melatonin values obtained hourly during the unmasking protocol (AUC, DLMO) were correlated with Tdrop (Tc_waking_ ‐ Tc_sleep_) and neurocognitive outcomes (*n* = 29) obtained ∼6 months later.

**Result:**

Greater melatonin AUC correlated with greater Tdrop during SD in males and females, however, correlations peaked at an earlier and more narrow range of Tdrop times in females (5–7pm, *r* = 0.40–0.46, *p* < 0.01) than males (5–10pm, *r* = 0.34–0.60, *p* < 0.001). In both sexes, earlier DLMO correlated with greater Tdrop (*p* < 0.05). During Recovery, AUC did not correlate with Tdrop in either sex, whereas DLMO still inversely correlated with Tdrop in males only (*p* < 0.01). Greater melatonin AUC correlated with immediate (*β* = .72, *p* = .003) and delayed recall memory (*β* = .71, *p* = .020) and visuospatial function (*β* = .67, *p* = .009) in females only. Greater Tdrop during SD was associated with executive function (*r* = 0.59–0.66) and attention (*r* = 0.57–0.67) in females and language in males (*r* = 0.51–0.67), all *p* <0.01.

**Conclusion:**

Melatonin release is strongly coupled to nocturnal Tdrop, however the timing and strength of this coupling exhibit sex differences. Greater melatonin and Tdrop may be protective for cognition particularly among female older adults. Future research in younger adults should determine whether aging‐related changes (e.g., menopause) influence observed sex differences.

